# Synthesis of fluorinated δ-lactams via cycloisomerization of *gem*-difluoropropargyl amides

**DOI:** 10.3762/bjoc.6.48

**Published:** 2010-05-14

**Authors:** Satoru Arimitsu, Gerald B Hammond

**Affiliations:** 1 Department of Chemistry, Kyoto University, Kyoto, Japan; 2 Department of Chemistry, University of Louisville, Louisville, Kentucky 40292, United States

**Keywords:** bicyclic lactams, cycloisomerization, difluoropropargyl, enyne, ring-closing metathesis

## Abstract

*gem*-Difluoro-1,7-enyne amides are suitable building blocks for the synthesis of difluorodihydropyridinones via a ring-closing metathesis reaction, and of 4,4-difluoro-3-oxoisoquinolines through a ring-closing metathesis–enyne metathesis tandem reaction. These products, in turn, undergo a Diels–Alder reaction to yield heterotricyclic systems in moderate to good yields.

## Introduction

It has been estimated that as many as 25% of all synthetic pharmaceutical drugs contain an amide bond [1]. Commonly, β- and γ-lactams are present in many natural products and pharmaceuticals, and the introduction of a *gem*-difluoromethylene moiety has been reported to improve their biological activities. For example, a *gem*-difluoro-γ-lactam can inhibit γ-lactamase, which is responsible for bacterial resistance to γ-lactam antibiotics [2–4]. Additionally, α,α-difluoro lactams are precursors of some biologically active compounds [5–8]. Our group’s entry in this arena started as a collaboration with Professor Fustero and resulted in the syntheses of fluorinated β- and γ-lactams [9–13]. This sparked our interest in the synthesis of larger-ring lactams, with six to eight members, because nitrogen-containing medium-size heterocyclics are found in many natural products as part of fused cyclic structures. In their pioneering work on middle-range lactams bearing fluorine(s), Fustero et al. developed a ring-closing metathesis of α,α-difluoro-1,*n*-dienyl amides to furnish the corresponding α,α-difluorinated lactams [14]. The synthesis of medium-size heterocycles by a metathesis reaction is quite relevant, as demonstrated by its extensive application to multi-fused heterocyclics [15–19]. We postulated that functionalized fluorinated enyne amides could be used for the synthesis of a chemically diverse suite of δ-lactams because enynes are suitable partners in ring-closing metathesis reactions or cycloisomerizations. An additional benefit of using enynes in metathesis reactions is that the resulting diene product could be further elaborated using a Diels–Alder reaction to construct bi- or tricyclic ring systems [20].

## Results and Discussion

Initially, we investigated the enyne metathesis reaction of fluorinated enyne **1a** with commercially available ruthenium carbene complexes, the Hoveyda–Grubbs second-generation catalyst being the most reactive (entries 1–3, Table 1). The reaction at 110 °C gave **2a-iso** as the major compound, probably through the isomerization of **2a** (entry 3, Table 1) [14]. The latter (**2a**) was isolated when the reaction was carried out at 70 °C in toluene (entry 4, Table 1). Other solvents did not give good yields or selectivities (entries 5 and 6, Table 1). From experimentation, it became clear that ethylene gas was crucial for driving this reaction forward (compare entry 4 with 7, Table 1) [21]. 2,6-Dichloro-1,4-benzoquinone, which has been reported to prevent isomerization [22], gave disappointing results (entry 8, Table 1). When our optimized conditions were applied to other fluorinated 1,7-enynes we isolated the desired lactams (entries 1–3, Table 2). Higher temperatures were required with internal alkynes (entries 2–5, Table 2), where isomerization occurs and the enyne ester **1d** did not yield satisfactory results. Interestingly, although enyne ketone **1e** gave a good ^19^F NMR yield (97%) of the desired diene **2e**, we could only isolate the *ortho*-fluorophenol **3** in good yield after silica gel chromatography. This unexpected result could have positive synthetic repercussions, as *ortho*-fluorophenol is a moiety that has attracted attention because it is present in some bioactive compounds [23–25].

**Table 1:** Screening reaction conditions for the enyne metathesis of **1a**.

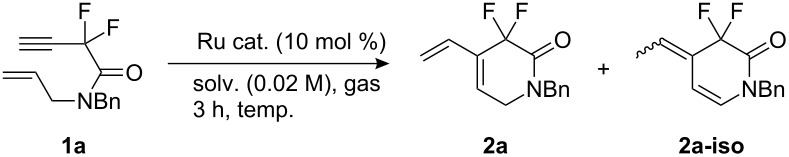					
Entry	Solvent	Ru cat.	Gas	Temp. (°C)	Yield of products (%)^a^ **1a/2a/2a-iso**

1	Toluene	G-I	C_2_H_4_	110	53/0/0
2	Toluene	G-II	C_2_H_4_	110	0/34/0
3	Toluene	HG-II	C_2_H_4_	110	0/6/66 (60)^b^
4	Toluene	HG-II	C_2_H_4_	70	0/85 (70)/0
5	1,2-DCE^c^	HG-II	C_2_H_4_	70	No rxn.
6	THF	HG-II	C_2_H_4_	70	30/25/0
7	Toluene	HG-II	Argon	70	28/34/0
8	Toluene	HG-II	C_2_H_4_^d^	110	0/20/11

^a^Yield was determined by ^19^F NMR and the value in parentheses is the isolated yield. ^b^**2a-iso** was isolated as an *E*/*Z* mixture (*E*/*Z* = 3/1). ^c^1,2-Dichloroethane. ^d^20 mol % of 2,6-dichloro-1,4-benzoquinone was used.

**Table 2:** Metathesis reaction of fluorinated 1,7-enyne carbonyl compounds.

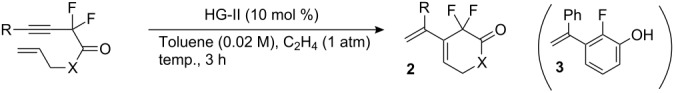				
Entry	X	R	Temp. (°C)	Yield of **2** (%)^a^

1	NBn	H (**1a**)	70	70 [85] (**2a**)
2	NBn	*n*-Hex (**1b**)	110	52 [78] (**2b**)
3	NBn	Ph (**1c**)	110	69 [95] (**2c**)
4	O	Ph (**1d**)	110	— [33]^b^ (**2d**)
5	C	Ph (**1e**)	110	— [97]^c^ (**2e**)

^a^The yields in brackets were determined by ^19^F NMR. ^b^Isolation of **2d** was unsuccessful due to the complex mixture that had been formed. ^c^Compound **3** was isolated in 84% after silica gel chromatography.

**Scheme 1:**
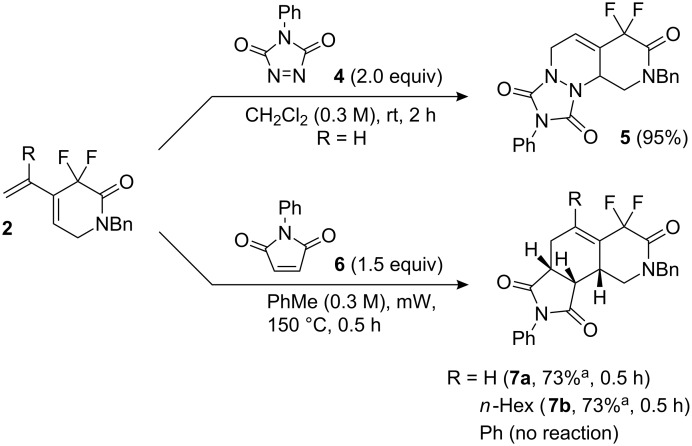
Diels–Alder reaction of diene **2** with **4** and **6**. ^a^The other isomers of **7a** and **7b** were isolated in 8% and 20% yield, respectively.

Dienes **2a** and **2b** were used in Diels–Alder reactions with **4** and **6** to produce **5** and 4,4-difluoroisoquinolin-3-one derivatives **7**, respectively, in excellent yield and good stereoselectivity (Scheme 1). Phenyl-substituted diene **2c** gave no reaction, even after a longer reaction time. The stereochemistry of **7a** and **7b** was determined by COSY and NOESY experiments.

**Scheme 2:**
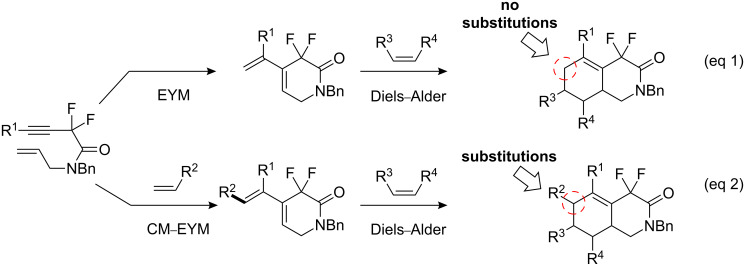
Synthetic concept toward multi-substituted *gem*-difluoroisoquinolinones.

Recently, various tandem reactions with ruthenium complexes have become popular in organic chemistry because Ru(II) complexes are capable of catalyzing additional reactions [26–27]. Since our enyne metathesis reaction of fluorinated 1,7-enynes does not permit substitution at the 6-position of the resultant *gem*-difluoroisoquinolinone (eq 1, Scheme 2), we examined a potential cross metathesis–enyne metathesis tandem-type reaction (CM–EYM reaction). In theory, if the terminal vinyl group of diene **2** can be modified by a tandem metathesis reaction, this would permit the synthesis of multi-substituted *gem*-difluoroisoquinolinones through a subsequent Diels–Alder reaction (eq 2, Scheme 2) [28].

In this regard, we screened various ruthenium carbene complexes using 1,7-enyne amide **1a** and styrene **8a** as a model reaction and found that the Hoveyda–Grubbs second-generation catalyst gave the best mass balance of products **2a** and **9a** (entry 3, Table 3). We obtained better results when the reaction was carried out in a sealed pressure reaction vessel (compare entries 3 and 4, Table 3). More interestingly, the choice of solvent had a tremendous effect on the selectivity between **2a** (EYM product) and **9a** (CM–EYM product) (entries 4–8, Table 3). Methylene chloride was found to be the best solvent (entry 5, Table 3). Other reaction factors were also examined carefully; higher concentrations reduced the yield and selectivity slightly (entries 9 and 10, Table 3). Lower reaction temperature (50 °C) resulted in no conversion (entry 11, Table 3), and the reaction produced a mixture of **2a** and **9a** in lower yield in the absence of ethylene gas (entry 12, Table 3).

**Table 3:** Screening of CM–EYM tandem reaction.

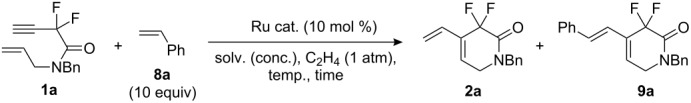						
Entry	Ru cat.	Solvent	Conc. (M)	Temp. (°C)	Time^a^ (h)	Yield of products **2a/3a** (%)^b^

1^c^	G-I	Toluene	0.02	110	1.5	Complex
2^c^	G-II	Toluene	0.02	110	1.5	23/17
3^c^	HG-II	Toluene	0.02	110	3	34/46
4	HG-II	Toluene	0.02	110	3	33/37
5	HG-II	CH_2_Cl_2_	0.02	110	24	0/68 (67)^d^
6	HG-II	1,2-DCE	0.02	110	24	26/24
7	HG-II	THF	0.02	110	24	4/28
8	HG-II	1,4-Dioxane	0.02	110	24	9/32
9	HG-II	CH_2_Cl_2_	0.05	110	24	0/56
10	HG-II	CH_2_Cl_2_	0.1	110	24	8/32
11	HG-II	CH_2_Cl_2_	0.02	50	24	No reaction
12^e^	HG-II	CH_2_Cl_2_	0.02	110	24	18/30

^a^Time was determined by TLC and/or GC–MS. ^b^The yield and ratio of products were determined by ^19^F NMR. ^c^The reaction was carried out without a pressure vessel. ^d^The value in parentheses is the isolated yield. ^e^The reaction was carried out under argon.

These optimized reaction conditions were applied to other vinyl compounds **8** (Table 4). After 4-substituted aryl alkenes gave the desired product **9** in moderate yields with excellent selectivity (*E*-major) (entries 3 and 4, Table 4), it then became clear that steric hindrance and the electronic deficiency of alkenes **8** decrease the efficiency of the tandem reaction; the non-tandem product **2a** being formed instead (entries 2 and 6, Table 4). Allyl acetate **8f** gave the desired product only when toluene was employed as solvent (entry 6, Table 4).

**Table 4:** CM–EYM tandem reaction with fluorinated 1,7-enyne **1a** and alkene **8**.

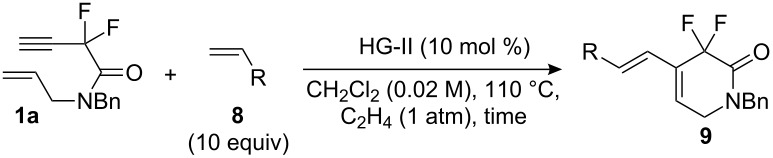			
Entry	R	Time (h)^a^	Isolated yields of **9** [*E*/*Z*]^b^ + **2a** (%)

1	Ph (**8a**)	24	67 [1/0] (**9a**) + 0
2	3-MeO-C_6_H_4_ (**8b**)	24	36 [1/0] (**9b**) + 15
3	4-MeO-C_6_H_4_ (**8c**)	24	33 [1/0] (**9c**) + trace
4	4-Cl-C_6_H_4_ (**8d**)	24	43 [1/0] (**9d**) + trace
5	4-F-C_6_H_4_ (**8e**)	27	33 [1/0] (**9e**) + 19
6^c^	CH_2_OAc (**8f**)	3	31 [1/0] (**9f**) + 31

^a^Time was determined by TLC and/or GC–MS. ^b^The ratio of products was determined by ^1^H and/or ^19^F NMR. ^c^Toluene was used instead of CH_2_Cl_2_.

**Figure 1:**
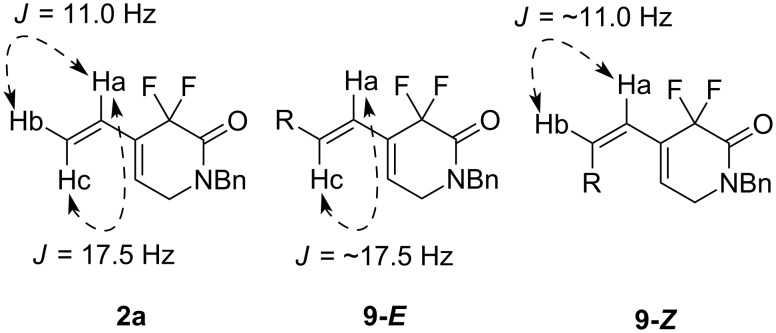
Comparison of coupling constant of vinyl protons.

The stereochemistry of the terminal double bond of **9** was determined by comparing coupling constants of vinyl protons of compound **2a**. The coupling constants of *trans*-protons (Ha–Hc) and *cis*-protons (Ha–Hb) on a double bond are *J* = 17.5 Hz and *J* = 11.0 Hz, respectively (Figure 1).

**Scheme 3:**
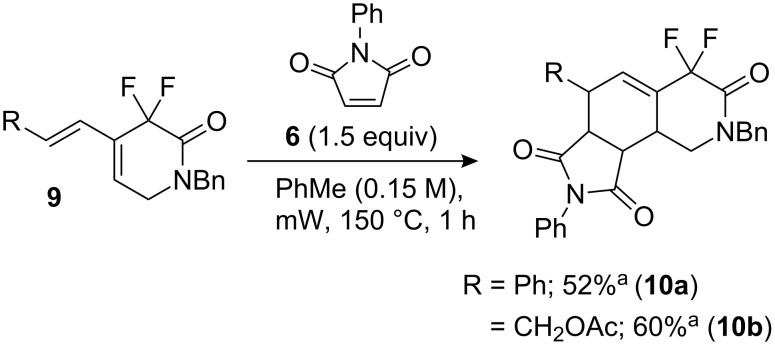
Diels–Alder reaction with **9** and **6**. ^a^Combined yield of two isomers.

As expected, the Diels–Alder reaction with *N*-phenylmaleimide **6** gave 6-substituted *gem*-difluoroisoquinolinones efficiently with slight stereoselectivity (Scheme 3).

In summary, *gem*-difluoro-1,7-enyne carbonyl derivatives are useful reaction partners in enyne metathesis cycloisomerization and CM–EYM tandem reactions catalyzed by ruthenium carbene complexes. The resulting diene products can be elaborated further using a Diels–Alder reaction.

## Supporting Information

File 1 Synthesis of fluorinated δ-lactams via cycloisomerization of *gem*-difluoropropargyl amides
