# Supplementary material for: Synthesis of fluorinated δ-lactams via cycloisomerization of gem-difluoropropargyl amides
Source: Beilstein J Org Chem. 2010 May 14;6:48. doi: 10.3762/bjoc.6.48 (PMC2887306; doi:10.3762/bjoc.6.48)
Supplement: File 1 — Synthesis of fluorinated δ-lactams via cycloisomerization of gem-difluoropropargyl amides [file Beilstein_J_Org_Chem-06-48-s001.pdf]

Supporting Information

for

Synthesis of fluorinated δ-lactams via cycloisomerization of *gem*-difluoropropargyl amides

Satoru Arimitsu[1] and Gerald B. Hammond*[2]

Address: [1]Department of Chemistry, Kyoto University, Kyoto, Japan and [2]Department of Chemistry, University of Louisville, Louisville, Kentucky 40292, United States

Email: Gerald B. Hammond - gb.hammond@louisville.edu

* Corresponding author

General information

^{1}H, ^{13}C and ^{19}F NMR spectra were recorded on a Varian Inova 500 instrument at 500, 126 and 470 MHz respectively. The deuterium solvents used for NMR are mentioned in the parentheses of their NMR assignments in the experimental section. The chemical shifts are reported in δ (ppm) values relative to the internal standard. Coupling constants are reported in Hertz (Hz). GC/MS analyses were performed on a Varian Saturn 2000 system. Infrared spectra were recorded using a Mattson Infinity Series FTIR or a Mattson Galaxy Series FTIR 5000 spectrometer. All air and/or moisture sensitive reactions were carried out under argon atmosphere. Solvents (THF, Et_2O, CH_2Cl_2, DMF) were dried using a PureSolv PS-400-4 purification system (Innovative Technology, Inc.). Benzene, toluene and 1,2-dichloroethane were distilled over CaH_2. 1,4-Dioxane was distilled over Na/benzophenone. All other reagents obtained from major chemical

suppliers were used without any purification. ^{19}F NMR yields in mixture were obtained using α,α,α-trifluoromethylbenzene or hexafluorobenzene as the internal reference. Silica gel column chromatography was performed using #R12030B, SiliaFlash® P60, 40–63 μm obtained from SiliCycle Inc. TLC was developed on Merck silica gel 60 F254 aluminum sheets. Prep. TLC was conducted on DC-fertigplatten SIL RP-18W/UV254 by Alltech Associates Inc. Elemental analysis was performed at Atlantic Microlabs Inc., Norcross, Georgia. HRMS was performed at Kyoto Institute in Japan.

The synthesis of **2**, **5** and **7** has already been described. See reference [1].

2-Fluoro-3-(1-phenylvinyl)phenol (3)

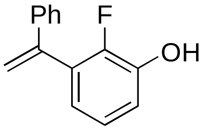

^{1}H NMR ($CDCl_3$) δ: 5.11 (d, *J* = 6.5 Hz, 1H), 5.35 (s, 1H), 5.37 (s, 1H), 6.73 (dt, *J* = 2.0, 7.0 Hz), 6.89–6.96 (m, *2*H), 7.21–7.25 (m, 5H); ^{19}F NMR ($CDCl_3$) δ: −142.11 (s, 2F); ^{13}C NMR ($CDCl_3$) δ: 116.5, 117.1, 122.6, 124.2 (d, *J* = 4.8 Hz), 126.8, 127.9, 128.3, 129.7 (d, *J* = 12.6 Hz), 140.4, 143.7, 143.8 (d, *J* = 15.4 Hz), 148.6 (d, *J* = 238.9 Hz); IR (neat) cm^{-1}: 3526, 3398, 3056, 2926, 1614, 1469, 1334, 1216; MS *m*/*z* (%): 214 (23, M^+), 193 (15), 165 (24); HRMS (FAB) calcd. for $C_{14}H_{11}FO$ (M^+): 214.0794, found: 214.0784.

1-Benzyl-3,3-difluoro-4-styryl-3,6-dihydro-1*H*-pyridin-2-one (9a)

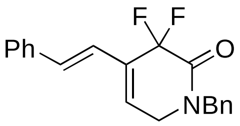

^{1}H NMR ($CDCl_3$) δ: 4.04 (bs, 2H), 4.77 (s, 2H), 6.26 (s, 1H), 6.68 (d, *J* = 16.5 Hz, 1H), 7.08 (d, *J* = 16.5 Hz, 1H), 7.29–7.40 (m, 8H), 7.47 (d, *J* = 7.5 Hz, 2H); ^{19}F NMR ($CDCl_3$) δ: −95.36 (s, 2F); ^{13}C NMR ($CDCl_3$) δ: 46.9, 50.0, 107.7 (t, *J* = 237.5 Hz), 120.6, 126.1 (broad d, *J* = 3.9 Hz), 128.2, 128.4 (overlapping 2C), 128.7, 129.0, 131.2 (t, *J* = 24.0 Hz), 133.2, 135.1, 136.3, 160.3 (t, *J* = 30.2 Hz).

1-Benzyl-3,3-difluoro-4-[2-(3-methoxyphenyl)vinyl]-3,6-dihydro-1*H*-pyridin-2-one (9b)

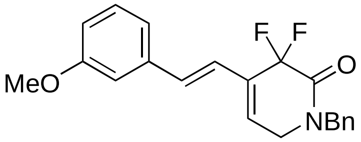

^{1}H NMR ($CDCl_3$) δ: 3.84 (s, 3H), 4.03 (s, 2H), 4.76 (s, 2H), 6.26 (s, 1H), 6.66 (d, *J* = 17.0 Hz, 1H), 6.85 (d, *J* = 8.0 Hz, 1H), 6.98 (s, 1H), 7.03–7.06 (m, 2H), 7.25–7.37 (m, 5H); ^{19}F NMR ($CDCl_3$) δ: −95.31 (s, 2F); ^{13}C NMR ($CDCl_3$) δ: 47.2, 50.3, 55.5, 108.0 (t, *J* = 237.5 Hz), 112.2, 114.4, 119.7, 121.2, 126.6, 128.5, 128.6, 129.2, 129.9, 131.4 (t, *J* = 25.4 Hz), 133.3, 135.3, 138.0, 160.1, 160.5 (t, *J* = 29.7 Hz).

1-Benzyl-3,3-difluoro-4-[2-(4-methoxyphenyl)vinyl]-3,6-dihydro-1*H*-pyridin-2-one (9c)

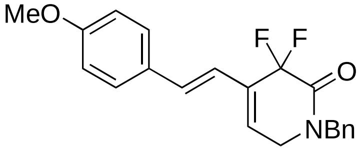

^{1}H NMR ($CDCl_3$) δ: 3.83 (s, 3H), 4.02 (bs, 2H), 4.75 (s, 2H), 6.20 (s, 1H), 6.54 (d, *J* = 16.5 Hz, 1H), 6.88 (d, *J* = 7.5 Hz, 1H), 7.02 (d, *J* = 16.5 Hz, 2H), 7.30–7.40 (m, 7H); ^{19}F NMR ($CDCl_3$) δ: −95.37 (t-like, *J* = 3.3 Hz, 2F); ^{13}C NMR ($CDCl_3$) δ: 46.9, 50.0, 55.3, 107.8 (t, *J* = 237.0 Hz),

114.1, 118.4, 125.11 (broad d, *J* = 3.9 Hz), 128.0, 128.2, 128.3, 128.9, 129.1, 131.7 (t, *J* = 24.4 Hz), 132.6, 135.1, 159.8, 160.3 (t, *J* = 30.0 Hz); IR (CCl_4) cm^{-1}: 3728, 2916, 2836, 2354, 2339, 1673, 1510, 1367, 1249.

1-Benzyl-3,3-difluoro-4-[2-(4-chlorophenyl)vinyl]-3,6-dihydro-1*H*-pyridin-2-one (9d)

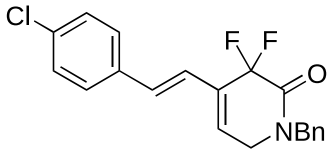

^{1}H NMR ($CDCl_3$) δ: 4.03 (bs, 2H), 4.78 (s, 2H), 6.25 (s, 1H), 6.63 (d, *J* = 17.0 Hz, 1H), 7.02 (d, *J* = 16.5 Hz, 1H), 7.82–7.38 (m, 9H); ^{19}F NMR ($CDCl_3$) δ: −95.34 (s, 2F); ^{13}C NMR ($CDCl_3$) δ: 46.9, 50.0, 107.6 (t, *J* = 237.6 Hz), 121.3, 126.7 (broad d, *J* = 3.8 Hz), 127.9, 128.5, 128.4, 128.9, 129.9, 131.0 (t, *J* = 24.3 Hz), 131.9, 134.1, 134.9, 135.0, 160.2 (t, *J* = 29.8 Hz); IR (CCl_4) cm^{-1}: 3063, 3034, 2942, 1895, 1684, 1490.

1-Benzyl-3,3-difluoro-4-[2-(4-fluorophenyl)vinyl]3,6-dihydro-1*H*-pyridin-2-one (9e)

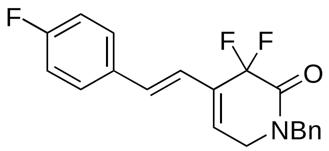

^{1}H NMR ($CDCl_3$) δ: 3.94 (m, 2H), 4.68 (s, 2H), 6.12 (s, 1H), 6.50 (d, *J* = 16.5 Hz, 1H), 6.93–6.98 (m, 3H), 7.19–7.36 (m, 7H); ^{19}F NMR ($CDCl_3$) δ: −95.35 (s, 2F), −113.27 (s, 1F); ^{13}C NMR ($CDCl_3$) δ: 46.9, 50.0, 107.7 (t, *J* = 237.6 Hz), 115.7 (d, *J* = 22.0 Hz), 119.1, 120.4, 126.3, 128.3 (d, *J* = 11.6 Hz), 128.4, 129.0, 131.1 (m), 131.9, 132.6, 135.1, 160.2 (t, *J* = 29.8 Hz), 162.8 (d, *J* = 248.5 Hz).

Acetic acid 3-(1-benzyl-3,3-difluoro-2-oxo-1,2,3,6-tetrahydropyridin-4-yl)allyl ester (9f)

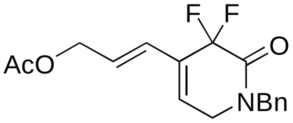

^{1}H NMR ($CDCl_3$) δ: 2.10 (s, 3H), 3.94–4.00 (m, 2H), 4.67 (d, J = 5.00 Hz, 2H), 4.74 (s, 2H), 6.25 (s, 1H), 6.17–6.30 (m, 3H), 7.29–7.39 (m, 5H); ^{19}F NMR ($CDCl_3$) δ: −95.78 (t-like, J = 6.6 Hz, 2F); ^{13}C NMR ($CDCl_3$) δ: 21.2, 47.0, 50.3, 64.5, 107.7 (t, J = 237.6 Hz), 125.6, 127.7 (broad d, J = 3.8 Hz), 128.5, 129.3 (overlapping 2C), 129.3, 130.5 (t, J = 24.5 Hz), 135.3, 134.1, 160.4 (t, J = 30.3 Hz), 170.9.

8-Benzyl-6,6-difluoro-2,4-diphenyl-4,6,8,9,9a,9b-hexahydro-3a*H*-pyrrolo[3,4-*h*]isoquinoline-1,3,7-trione (10a)

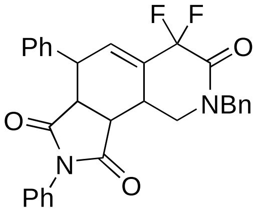

An oven-dried microwave vial (10 mL size) equipped with a stirring bar and under an argon atmosphere was charged with compound **9a** (1.0 equiv) and **6** (1.5 equiv) in toluene (0.15 M). The vial was then capped under argon and placed in a CEM Discover microwave synthesizer at 115 °C for 1 h (at 150 W, 250 psi max), and the temperature was monitored by computer during the reaction. After cooling to room temperature, the solvents were removed by a rotary evaporator. The residue was purified on a silica gel column chromatography to afford the compound **10a** as a liquid.

^{1}H NMR ($CDCl_3$) δ: 2.47 (t, J = 10.0 Hz, 1H), 2.56 (bs, 1H), 3.08 (t, J = 9.5 Hz, 1H), 3.52 (bs, 1H), 3.84 (dd, J = 14.0, 5.0 Hz, 1H), 3.89 (dd, J = 13.5, 5.0 Hz, 1H), 4.57 (d, J = 14.0 Hz, 1H), 4.69 (d, J = 14.5 Hz, 1H), 6.63 (bs, 1H), 7.12–7.34 (m, 15H); ^{19}F NMR ($CDCl_3$) δ: −104.52 (d, J

= 271.1 Hz, 1F), −109.99 (d, *J* = 270.6 Hz, 1F); ^{13}C NMR ($CDCl_3$) δ: 34.0, 41.4, 42.0, 45.9, 47.1, 51.0, 109.2 (t, *J* = 245.6 Hz, 1H), 126.1, 127.6, 128.1, 128.2, 128.6, 128.8, 129.0, 129.0 (overlapping 2C), 129.2, 131.1, 131.6 (t, *J* = 20.3 Hz, 1H), 135.6, 141.8, 161.9 (t, *J* = 30.2 Hz, 1H), 175.5, 175.8.

Acetic acid 8-benzyl-6,6-difluoro-1,3,7-trioxo-2-phenyl-2,3,3a,4,6,7,8,9,9a,9b-decahydro-1*H*-pyrrolo[3,4-*h*]isoquinolin-4-ylmethyl ester (10b)

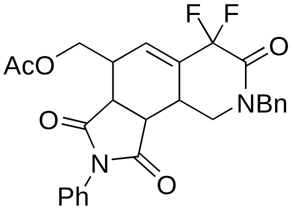

The compound **10b** was synthesized by the same procedure used for compound **10a**.

^{1}H NMR ($CDCl_3$) δ: 1.18 (t, *J* = 6.0 Hz, 1H), 2.02 (s, 3H), 2.64 (bs, 1H), 2.70 (bs, 1H), 3.27 (t, *J* = 8.0 Hz, 1H), 3.45 (t, *J* = 8.0 Hz, 1H), 3.53 (dd, *J* = 14.0, 6.0 Hz, 1H), 3.40 (dd, *J* = 7.8, 4.0 Hz, 1H), 4.13 (d, *J* = 14.5 Hz, 1H), 4.57 (t, *J* = 10.0 Hz, 1H), 4.71 (dd, *J* = 11.5, 6.5 Hz, 1H), 5.08 (d, *J* = 14.5 Hz, 1H), 6.58 (s, 1H), 7.02 (d, *J* = 8.0 Hz, 2H), 7.18–7.41 (m, 8H); ^{19}F NMR ($CDCl_3$) δ: −103.06 (d, *J* = 271.1 Hz, 1F),−112.97 (d, *J* = 271.1 Hz, 1F); ^{13}C NMR ($CDCl_3$) δ: 20.9, 33.9, 36.2, 42.0, 42.3, 43.9, 50.7, 63.6, 108.5 (t, *J* = 246.2 Hz), 126.5, 128.06, 128.12, 128.9, 129.2, 129.4, 131.1, 131.8, 132.3 (t, *J* = 21.4 Hz), 135.4, 161.7 (t, *J* = 29.7 Hz), 170.7, 174.7, 174.9.

Important cross peaks in COSY of **7a**.

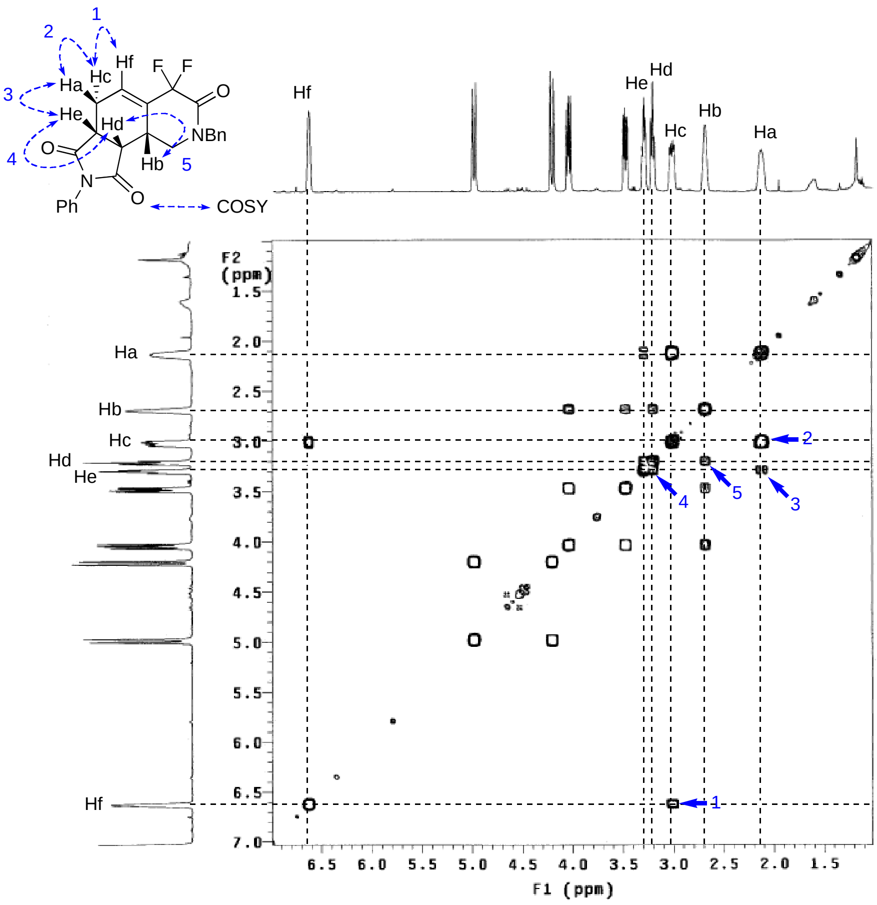

Important cross peaks in NOESY of **7a**.

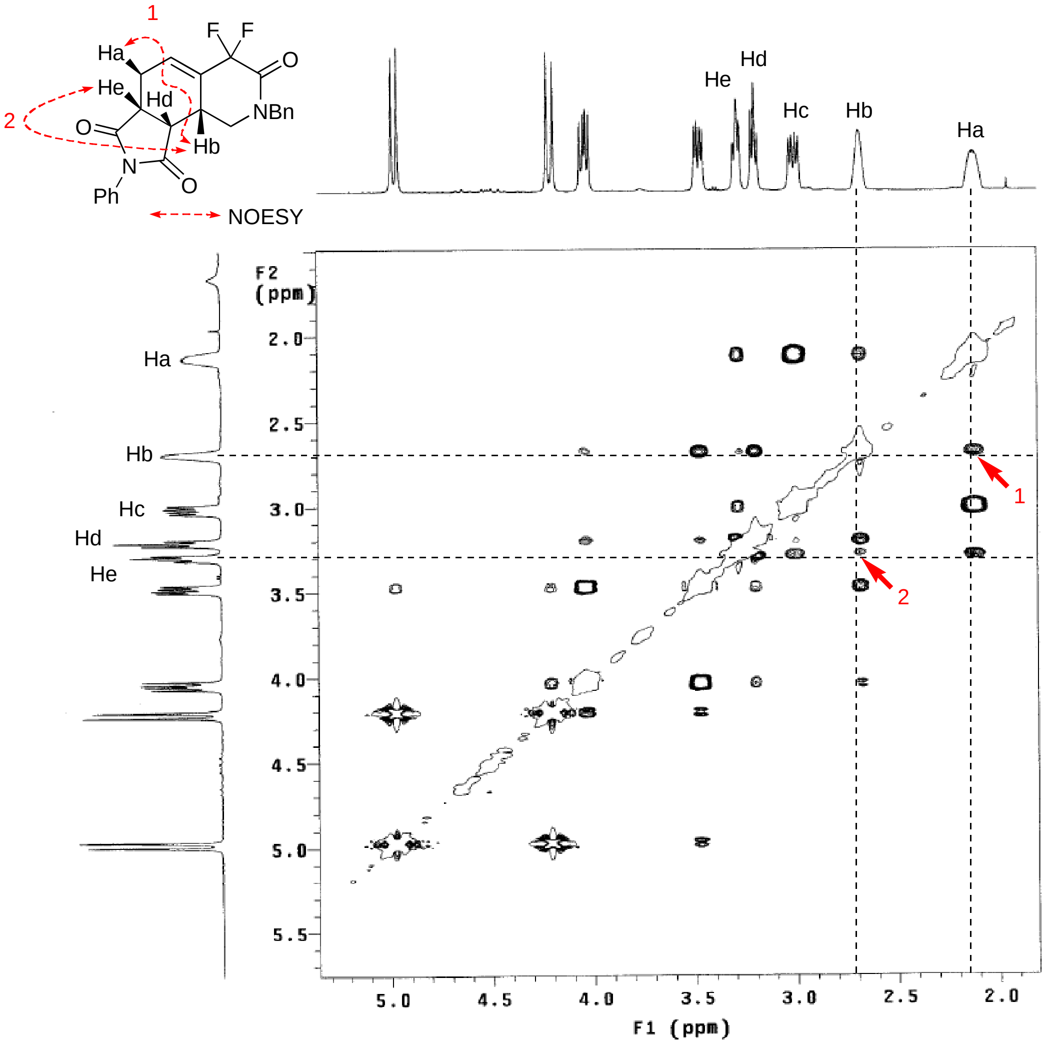

References

1. Arimitsu, S.; Fernández, B.; del Pozo, C.; Fustero, S.; Hammond, G. B. *J. Org. Chem.* **2008,** *73,* 2656–2661. doi:10.1021/jo7025965
